# Characterising ‘obesogenic’ versus ‘protective’ food consumption and their value chain among Ghanaian households

**DOI:** 10.1017/S1368980025000114

**Published:** 2025-02-06

**Authors:** Reginald Adjetey Annan, Nana Ama Frimpomaa Agyapong, Robert Aidoo, Charles Apprey, Linda Nana Esi Aduku, Elizabeth C Swart

**Affiliations:** 1 Department of Biochemistry and Biotechnology, College of Science, Kwame Nkrumah University of Science and Technology (KNUST), PMB University Post Office, Kumasi, Ghana; 2 Department of Clinical Nutrition and Dietetics, College of Health and Allied Sciences, University of Cape Coast, Cape Coast, Ghana; 3 Department of Agricultural Economics, Agribusiness & Extension, Faculty of Agriculture, Kwame Nkrumah University of Science and Technology, Kumasi Ghana; 4 Office of Grants and Research, College of Science, Kwame Nkrumah University of Science and Technology (KNUST), PMB University Post Office, Kumasi, Ghana; 5 Department of Dietetics and Nutrition, University of the Western Cape, South Africa

**Keywords:** Nutrition transition, Obesogenic food environment, Ghana

## Abstract

**Objectives:**

This article explores the characteristics of Ghanaian households’ consumption of obesogenic *v*. protective foods, including their retail, distribution and origin.

**Design:**

A household food consumption survey was conducted using an adapted Prospective Urban and Rural Epidemiology study FFQ. Product pathways for selected obesogenic (processed meat, sugar-sweetened beverages (SSB) and biscuits) and protective (cooked vegetables, legumes and fish) foods were traced from retailers through distributors/wholesalers to producers.

**Setting:**

Rural and urban communities in the Ashanti Region and selected retail/wholesale/producers nationwide.

**Participants:**

612 households, 209 retailers and 185 wholesalers/distributors.

**Results:**

About 20 % of households consume SSB and confectionery weekly, and just 2 % consumed processed meat. Of the protective foods, fish had the highest proportion of households consuming weekly (74·5 %), followed by cooked vegetables (53·1 %) and legumes (22·8 %). Frequent SSB consumption is higher in younger (*P* < 0·001), male (*P* = 0·010), urban (*P* < 0·001) and more educated (*P* < 0·001) food purchaser households. Below 10 % of households followed the healthiest dietary pattern (high-protective-and-low-obesogenic) but higher in older and more educated food purchaser households. In contrast, most households (about 80 %) consumption patterns did not discriminate between obesogenic and protective foods. Generally, characteristics of purchasers from retail/wholesale outlets agree with those of households, where obesogenic foods were retailed to younger, less educated buyers than older, more educated ones. While the protective foods had a strong local producer presence, the obesogenic foods were predominantly imported.

**Conclusion:**

Household consumption and retail/distribution of obesogenic foods are associated with socio-demographic characteristics, but obesogenic foods are almost entirely produced outside Ghana. Policies that regulate importation on health grounds can promote a healthier food environment.

Sub-Saharan African countries such as Ghana are experiencing a double malnutrition burden, where both undernutrition and overweight/obesity coexist. While childhood undernutrition manifested by stunting, wasting and underweight among children and micronutrient deficiencies in children and women persist^(1)^, in the past few decades, non-communicable diseases (NCD) prevalence among adults has increased significantly^(2)^. Evidence shows that overweight and obesity, drivers of NCD among adults, are on the rise in the country^(1,3)^, and these are associated with consuming high amounts of calories, energy-dense foods and low physical activity^(4)^. The UN has projected that the global economic loss due to NCD can reach $47 trillion by 2030, while the total number of deaths from NCD can reach 52 million by this same year^(5)^. This makes addressing the drivers of NCD in the country a significant step towards economic growth and development.

The obesity epidemic experienced in Ghana and many low-and-middle-income countries (LMIC) have been attributed to nutrition transition, defined as modernisation, urbanisation, economic development and increased wealth that a country experiences. The aforementioned leads to predictable shifts in diets to more energy-dense foods and low levels of physical activity^(6)^. The nutrition transition has been described as a phenomenon that is too simple, but it explains in detail the five periods of dietary transition that populations undergo^(7,8)^. Many LMIC, including Ghana, are at the fourth stage of the nutrition transition. This stage is characterised by increased access and/or consumption of high-calorie, high-fat, sugar and refined carbohydrate foods while physical activity has reduced^(4)^. Their consequences include increased obesity-related chronic diseases, like diabetes and heart disease. The nutrition transition is thus the driver of the shift to diseases associated with high caloric diet and physical inactivity that LMIC, including Ghana, currently face^(9)^.

The stages of the nutrition transition experienced decades ago by developed countries are said to be remarkably different from current experiences in LMIC^(10)^. The developed countries experienced a total shift from undernutrition to overweight and obesity, whereas, for most LMIC, both undernutrition and overweight/obesity co-exist^(10)^. The timing and rate of the change are different, and at the same time, the politics and capacity of LMIC to address the problem differ^(11)^. This calls for a better understanding of the drivers of these differences.

Several recent epidemiological studies correlating dietary consumption with obesity and NCD risk indicate that the consumption of processed foods like potato chips, fried potatoes, SSB, processed meat, unprocessed red meats, sweets and desserts, butter or margarine and refined grains are associated with increased risk of obesity and related NCD^(12–15)^. Obesogenic food environments, which refer to surroundings, opportunities and conditions that promote the consumption of foods that increase an individual’s or population’s risks for obesity, have been linked to increasing obesity in populations^(16)^. Current food systems or the activities, actors and networks that interact along and around the whole food value chain, including production, processing, distribution, consumption, waste disposal and recycling,^(17)^, drive increasing availability and access to foods that increase obesity risk.

In response to the increasing prevalence of obesity and associated nutrition-related NCD globally^(18)^, there are calls to improve the food environment and promote healthier foods^(19)^. Yet, a better understanding of the nature and drivers of food environments will enable appropriate policies to curb this menace. The Researching the Obesogenic Food Environment (ROFE) project was to understand better the nature of the food environment and the drivers of the changing nature of marketing in urban and rural communities in South Africa and Ghana^(20)^. A better understanding of the food environment will help identify the potential policy levers available to improve the healthfulness of the local food environment. Initial findings of the ROFE study showed that neighbourhood food availability measured by retail outlets was associated with consumption^(21)^, implying that the food environment drives the foods consumed.

Population dynamics, such as age, gender, income, education and occupation and urbanisation, can drive the acquisition and consumption of obesogenic foods^(22)^. For example, urbanisation can drive economic development through better education, occupation and increased income. Still, it may also increase poverty through the influx of rural folks to urban areas without consideration of jobs, limiting food access and promoting energy-dense food intake^(22)^. This paper describes the socio-demographic characterization of the consumption, retail and wholesale/distribution of obesogenic *v*. healthy foods among Ghanaian households. We also identify countries or origins of obesogenic *v*. healthy foods.

A better understanding of the characteristics of household obesogenic and healthy foods consumption in LMIC such as Ghana provides data for identifying possible policy targets to address NCD and their related economic, health and social impacts^(23–25)^. NCD strain healthcare systems with high treatment costs and reduce productivity due to illness-related absenteeism^(23)^. They often lead to long-term health complications, increasing the financial burden on individuals and families^(23)^. NCD contribute to a decline in overall well-being, reducing life expectancy and quality of life and hence negatively impacting the health of populations^(24)^. Socially, NCD can exacerbate inequalities, as marginalised communities often face barriers to accessing healthcare services such as health education, leading to disparities in disease prevalence and outcomes^(25)^.

## Methods

**Figure 1.**
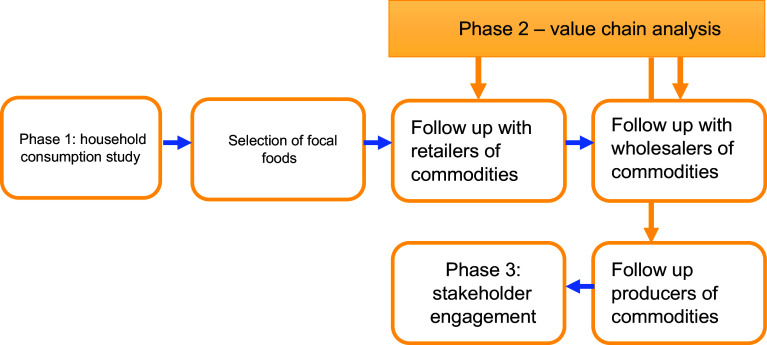
Study phases.

Figure 1 provides the flow diagram of the study. The ROFE study was implemented in three phases described in detail elsewhere^(21)^ and briefly described in the sections below. The study was implemented in three stages: phase 1 determined household food consumption and related factors. Phase 2 assessed the value chains of healthy and obesogenic food commodities. In contrast, phase 3 utilised a political economy analysis approach to elicit data to develop more robust policies to promote nutrition and support other food system objectives^(20)^. For this study, we report on data collection only in Ghana to characterise factors associated with household food consumption and value chain (retail, distribution and production) of obesogenic and healthy foods.

### Study sites in Ghana

The ROFE study was conducted in South Africa and Ghana. In both countries, a rural and urban community was chosen for the household food consumption study to enable assessment of how different these contexts are as far as household consumption of obesogenic *v*. healthy foods and their determinants are concerned. Ahodwo is a suburb in Kumasi, the capital city of Ashanti Region Ghana, while Ejuratia is a rural community in the Kwabre East District of Ashanti Region. Ahodwo was, therefore, selected to represent an urban community, while Ejuratia was chosen to represent a rural community. These sites were chosen because they were viable for interrogating the variables of interest and the researchers have experience in working at these study sites. The value chain analysis involved tracking foods identified in the consumption studies across the country’s retail, wholesale and production systems.

### Data collection

#### Household socio-demographic characterization

The ODK smartphone survey app was used to collect data from digital survey instruments. A structured questionnaire was developed in a consultative process involving the entire interdisciplinary research team. The instrument incorporated standardised survey instruments such as the Lived Poverty Index^(26)^ and other demographic characteristics like age, sex, educational level, income and marital status of the primary decision maker for household food acquisition. Enumerators reviewed and validated the questionnaires as part of an iterative training process, including several workshops to ensure clarity and consistency of comprehension. The survey is described in detail elsewhere^(21)^. The Lived Poverty Index is an experiential measure based on a series of survey questions about how frequently people go without necessities such as food, fuel, and medicine. The Lived Poverty Index was used to classify households as deprived or not deprived. For this study, households were grouped as deprived if they reported having gone without these necessities due to lack of access and not by choice.

#### Phase 1- Household food consumption survey

A household food consumption survey was undertaken in 612 households. Households in this study were selected using a systematic random sampling approach. In Ahodwo, the town was divided into six sections based on the main streets. Starting at a randomly chosen point on each street, every fifth household was selected for the study. In Ejuratia, the main lorry station served as the central point, from which the town was divided into four sections. After a random start in each section, every third household was selected. An adapted version of the Prospective Urban and Rural Epidemiology was used to collect data on the usual food consumption of households^(21)^. The questionnaire comprised a list of commonly consumed food items for respondents to indicate their frequency of consumption by the household. The adult man or woman in charge of or knowledgeable about food acquisition for the household was asked to answer questions on the household’s food acquisition decision-making and consumption. The FFQ data from the household food consumption study were used to categorise household food consumption patterns into ‘obesogenic’ or ‘protective’. The nutritional classification framework used to do this classification was informed by the NOVA system and deliberation among the authors. Food groups were allocated to two classes of obesity risk – obesogenic *v*. protective – based on their classification in terms of the NOVA system. The NOVA classification system categorises foods based on the extent and purpose of their processing. It divides food into four groups: unprocessed or minimally processed foods, processed culinary ingredients, processed foods and ultra-processed food and drink products, providing a framework to assess the impact of food processing on nutritional quality^(27)^. For this study, foods on the FFQ where the evidence for impacts on obesity was clear and compelling were considered. Other foods, where the evidence is more ambiguous (e.g. maize meal, red meat, and chicken), were not counted towards the obesity risk index. Through this process, minimally processed or plant-based foods on the FFQ were categorised as protective, while ultra-processed, processed and fried foods and ingredients on the FFQ were classified as obesogenic (Table 1).

**Table 1.** Categorisation of foods into obesogenic and protective Table 1 long description.

Categories	Definition and description
Obesogenic foods	Ultra-processed, processed and fried foods and ingredients. List of foods: Processed meat; instant noodles; salty snacks; sugary drinks; ready-to-eat foods; fast food; fried potatoes/hot chips; processed dairy; breakfast cereals; sweets; confectionery; sugar; Vetkoek/dumpling; commercial bread—White; commercial Bread—brown)
Protective foods	Minimally processed, plant-based foods. List of foods: Vegetables—fresh; vegetables—cooked; vegetables (fried/stir fry); fruit; legumes; bread—wholewheat; Fish

#### Determination of associations between socio-demographic characteristics of the decision maker for food acquisition and household food consumption

The FFQ had four frequency of consumption categories: daily, weekly, monthly and occasional/never. To allow a 2 by 2 analysis using the *χ* ^2^ (Fisher exact) test, the daily and weekly categories were combined into one group and the monthly and occasional/never into another group. From the household food consumption survey, we selected the three most consumed protective and three most consumed obesogenic foods to relate to the socio-demographic characteristics of the households. The selection of the three most consumed foods allowed for a value chain analysis of these foods and to predict the value chain of similar foods to determine which aspects of supply policies can be targeted. A discussion was held amongst team members, and three foods each were selected from the protective and obesogenic categories. This was to ensure detailed value chain analysis also based on project resources.

We determined the association between the proportions of frequently (weekly) and infrequently (less than weekly) consuming households of the three obesogenic and protective foods by age group, sex, occupation type, income level, education and urbanisation status of the individual in charge of food consumption decision within the household.

#### Determination of household obesogenic and protective food consumption patterns

All foods on the FFQ were used to determine households’ pattern of consuming obesogenic or protective food. A frequency cut-off was set to establish whether a given food is consumed frequently enough to contribute to obesity risk or prevention. For the obesogenic foods, two or more occasions of consumption per week for four foods in the category was selected to reflect the threshold for considering a household as consuming an obesogenic pattern. For the protective foods, five or more occasions of consumption per week of three foods in that category was selected as the threshold for protective consumption. These definitions and classifications are shown in Table 2. Using this classification, households that reported consuming processed meat, industrial bread, cookies and SSB at least twice a week, for example, would have reached the cut-off for obesogenic consumption. Households that consumed fruits, cooked vegetables, legumes (cowpea) and leafy green vegetables (cocoyam leaves of *Kontomire*) on five occasions in a week would have reached the minimum cut-off to be classed protective. Through this classification, households could consume both obesogenic and protective foods above the cut-off, only obesogenic food above the cut-off and only protective foods below the cut-off, protective foods above the cut-off and obesogenic foods below the cut-off or consume both categories of foods below the cut-offs. This allowed for the determination of four dietary patterns related to obesity or protection from obesity:

1. High obesogenic and high protective dietary pattern
2. High obesogenic and low protective dietary pattern
3. High protective and low obesogenic dietary pattern
4. Low obesogenic and low protective dietary pattern

**Table 2.** Definition of protective *v*. obesogenic food patterns Table 2 long description.

Categories	Definition and description
Consumption of protective foods by households	Frequently is defined as 5 or more times (occasions) per week for three of the protective foods
Consumption of obesogenic foods by households	Frequently is defined as consuming 2 or more times per week for four of the obesogenic foods
Names of patterns	
High obesogenic and high protective pattern	Households consume both obesogenic and protective foods frequently
High protective and low obesogenic pattern	Households consume protective foods frequently and obesogenic foods infrequently
High obesogenic and low protective pattern	Households consume obesogenic foods frequently and protective foods infrequently
Low obesogenic and low protective dietary pattern	Households consume both obesogenic and protective foods infrequently

From these patterns, households that followed the ‘high protective and low obesogenic’ dietary pattern would have enough frequency of protective foods and below the cut-off for obesogenic foods, making that pattern the ‘healthiest’. Households that followed the ‘high obesogenic and low protective’ dietary pattern were the worst as they consumed obesogenic foods above the cut-off but below the cut-off for protective foods. The ‘high obesogenic and high protective’ dietary pattern households consumed both categories above the cut-offs, meaning such households had protection from protective foods and obesity risk from the obesogenic foods at the same time, while the ‘low obesogenic and low protective’ dietary pattern households would be below the cut-off defined for both obesogenic and protective foods, implying that they neither had enough protection from obesity nor risk.

#### Determination of associations between household obesogenic or protective food consumption patterns and socio-demographic characteristics of household food purchaser

Chi-square analysis was used to determine associations between household consumption patterns and socio-demographic characteristics of the person in charge of household food acquisition.

### Phase 2

#### Selection of focal foods for value chain study

The three most consumed obesogenic and the three most consumed protective foods identified from the household food consumption study were used for the value chain analysis. The three protective foods were fish, legumes and cooked vegetables, while the obesogenic foods were SSB, processed meat and confectionery. However, for the value chain, cowpea was chosen for the legume category, and cocoyam leaves (*Kontomire*) were selected for the cooked vegetables category. For the obesogenic category, biscuits/cookies were selected as the confectionery item for the value chain analysis. For the legume category of the protective foods, legume was chosen because it is the most consumed legume in Ghana. Kontomire was also selected for the cooked vegetable category because it is commonly consumed in both study sites. Biscuits/cookies were selected as the food items for the obesogenic food category for the value chain analysis under the confectionery category. Ghanaian households frequently consume all the foods chosen for the value chain analysis.

#### Value chain analysis

The value chain analysis involved tracing these foods backwards from the retail outlets where households bought them, through the wholesale or distributors the retail outlets sourced them from, to the producers or countries of origin of the food commodities. A structured questionnaire was used to elicit relevant information from the target groups, and snowballing was used to identify the people. The questionnaire was administered to the retailers, wholesalers and producers to elicit details on the characteristics of households that bought from them.

#### Selection of retail outlets

**Figure 2.**
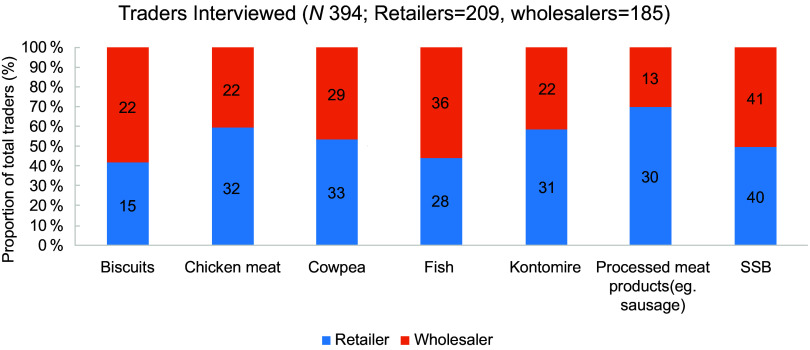
Characteristics of traders interviewed.

Households’ responses on where they bought food and the researchers’ local knowledge helped identify retailers for each food commodity. At least 30 retailers were determined for each food commodity in the communities where the household food consumption studies were carried out. In all, 209 retailers were interviewed (Figure 2).

#### Selection of wholesale (distributors) of obesogenic *v*. protective foods

The retailers for each food commodity mentioned the distributors/wholesalers whom they bought from for follow-up. Once those wholesalers were interviewed, snowballing was used to identify other wholesalers/distributors until 30 wholesalers were recruited for each commodity or there were no leads to more of them. In all, 185 wholesalers were interviewed.

#### Selection of producers for obesogenic *v*. protective food commodities

The wholesalers/distributors recruited for each food commodity led the researchers to the producers of the commodity or the countries they imported from. Some wholesalers/distributors sourced from other wholesalers/distributors while others sourced directly from producers or countries of origin of the food commodities.

#### Data collection from retailers, wholesalers (distributors) and producers

The structured questionnaire used for the survey of retail outlets, wholesale and producers of the food commodities allowed the collection of information to:

- Characterise the demography of households (in terms of income level, educational level, occupation type, sex, age and location) who purchased obesogenic *v*. healthy foods from retail shops, wholesalers/distributors and producers.
- Determine the movement of healthy *v*. obesogenic foods, including the countries from which they came and the types of organisations involved in their distribution.

### Data analysis

Statistical Package for Social Sciences (SPSS) version 21 and Excel were used for statistical analysis. Data are presented using basic frequencies and proportions to compare the characteristics of households consuming obesogenic *v*. healthy foods and the pattern of obesogenic *v*. healthy diets. The data from the value chain are presented in simple frequencies and proportions to characterise the demography of households who purchased from retailers, wholesalers, and producers. The chi-square test and Fisher‘s exact test were used to determine the significance of associations where necessary.

## Results

### Characteristics of households in food consumption survey

The consumption study included 612 households. A higher proportion of the respondents (43·7 %) were between 36 and 60 years, followed by 40·6 % below 36 years. Close to a third of households were deprived using Lived Poverty Index, while close to four in ten household respondents earned <100 USD (< 500 cedis at the time of the study) per month. Table 3 provides full details of the socio-demographic characteristics of household respondents.

**Table 3.** Socio-demographic characteristics of household food consumption decision maker Table 3 long description.

Variable	Frequency	%
Age in years		
< 36	248	40·6
36–60	267	43·7
> 60	96	15·7
Sex		
Male	114	18·6
Female	498	81·4
Community		
Rural	304	48·7
Urban	320	51·3
Lived poverty index		
Non-deprived	412	67·9
Deprived	195	32·1
Income		
< 500	166	38·1
500–1265	200	45·9
> 1265	70	16·1
Education		
None	121	19·8
Primary	63	10·3
Junior Secondary	190	31·0
Senior Secondary	167	27·3
Tertiary	71	11·6

10 Ghana cedi equivalent to 1 USD.

### Characteristics of households consuming obesogenic and protective foods

Table 4 shows the consumption of three obesogenic food commodities (SSB, processed meat and confectionery) and protective foods (cooked vegetables, legumes and fish) by socio-demographic characteristics of households. For the obesogenic food commodities, 19·5 % of the households reported consuming SSB at least once a week, while 20·9 % consumed confectionery at least once a week. Only 2 % of households reported consuming processed foods at least once a week.

**Table 4.** Proportions of households consuming selected protective and obesogenic food commodities weekly by household food consumption decision maker socio-demographic characteristics Table 4 long description.

Proportion consuming food groups weekly (%)							
Sociodemographic factors		SSB	Processed meat	Confectionery	Cooked vegetables	Legumes	Fish
(%)	Overall proportion	19·5 %	2·0 %	20·9 %	**53·1 %**	**22·8 %**	**74·7 %**
Age (%)	< 36	29·8	4·0	25·4	56·0	26·2	70·6
	36–60	17·0	0·8	15·1	57·0	21·1	78·9
	> 60	11·6	1·1	22·1	46·3	21·1	74·7
	*P*-value	**0·001**	**0·034**	**0·014**	0·181	0·342	0·096
Sex (%)	Male	30·4	2·7	20·5	48·2	29·5	74·1
	Female	19·3	2·0	20·5	56·5	21·7	75·3
	*P* value	**0·010**	0·716	0·998	0·110	0·080	0·800
Community (%)	Urban	29·4	2·3	24·2	48·4	21·6	75·5
	Rural	13·2	2·0	16·8	61·8	24·7	74·3
	*P* value	**< 0·001**	0·784	**0·023**	**< 0·001**	0·363	0·744
LPI (%)	Non-deprived	23·6	2·9	22·1	54·0	26·5	70·6
	Deprived	16·9	0·5	15·9	56·9	15·9	83·6
	*P* value	0·061	0·071	0·073	0·501	**0·004**	**0·010**
Income (%)	< 500 (< 66 USD)	16·4	0·6	16·4		23·6	74·5
	500–1265 (66–166·5 USD)	25·5	2·5	21·0		21·0	78·0
	> 1265·(> 166·5)	230·0	2·9	20·0	30·0	22·9	75·7
	*P* value	0·340	0·267	0·521	**< 0·001**	0·828	0·736
Education (%)	None	8·3	0·0	9·9	53·7	20·7	72·7
	Primary	17·7	1·6	14·5	50·0	21·0	62·9
	Junior Secondary	20·0	2·6	19·5	55·3	23·7	77·4
	Senior Secondary	28·3	1·8	28·3	57·8	22·3	78·3
	Tertiary	34·3	5·7	28·6	54·3	30·0	75·7
	*P* value	< 0·001	0·099	0·001	0·867	0·639	0·150

*n* 608. *χ* ^2^ and Fisher exact was used to determine the significance of associations between consumption of proportions and socio-demographic factors. Proportions presented are for households consuming the food every week. Households consuming less than weekly are not presented to decongest the table. Therefore, if 19·5 % consume SSB weekly means 81·5 % consume less than weekly (monthly to occasionally), adding up to 100 %. Boldface indicates statistically significant results.

SSB, sugar-sweetened beverage.

Frequencies of consumption of obesogenic foods were higher in households where the food purchaser was younger, in an urban area and more educated. Proportions of households consuming SSB at least every week was associated with lower age household respondents (*P* < 0·001), higher among male than female respondents (*P* = 0·010), higher among urban than rural households (*P* < 0·001) and higher in educated respondents (*P* < 0·001). No statistical differences were observed between deprived and non-deprived households (*P* = 0·061). Similarly, the frequency of consumption of confectionery was higher with higher age (*P* = 0·014), higher among urban than rural households (*P* = 0·023) and increased with the education level of household food purchasers (*P* < 0·001). Consumption frequency of processed meat showed a significant association with only age, where frequency reduced with age.

Among the three protective food commodities, fish was the most frequently consumed (weekly) among households (74·7 %), followed by cooked vegetables (5·1 %), and legumes (22·8 %).

From Table 4, consumption of fish and legumes did not differ by any socio-demographic characteristic, except by Lived Poverty Index, where more deprived (83·6 %) than non-deprived (70·6 %) households consumed fish at least every week (*P* = 0·001). On the other hand, weekly consumption of legumes was higher in non-deprived households (26·5 %) than in deprived households (15·9 %) (*P* = 0·004). The frequency of consumption of vegetables differed by income and community type. Weekly consumption of cooked vegetables was more frequent among rural households (61·6 %) compared to urban ones (41·8 %) (*P* < 0·001) and more frequent among households in the upper third of income (monthly income above 166·5 USD) (70 %), compared to the lower (< 66 USD) (39 %) and middle (66–166·5 USD) (43 %), (*P* < 0·001) class.

### Household obesogenic *v*. protective dietary pattern by socio-demographic characteristics

Table 5 shows a comparison of household dietary intake patterns by the four patterns. The majority (close to 80 %) of households either consumed both obesogenic and protective foods frequently (high obesogenic and high protective dietary pattern) or both groups infrequently (low obesogenic and low protective dietary pattern). The proportion of households that followed the high protective and low obesogenic dietary pattern was <10 %. The majority of the households were not on the extreme ends of following mainly protective dietary patterns or obesogenic dietary patterns.

**Table 5.** Socio-demographic characteristics of household food consumption decision maker by patterns of household food consumption Table 5 long description.

	Variable	Total	High protective and high obesogenic dietary pattern		High obesogenic and low protective pattern		Low obesogenic and low protective dietary pattern		High protective and low obesogenic dietary pattern		*P* value
			*n*	%	*n*	%	*n*	%	*n*	%	
Age	< 36	292	175	59·9	20	6·8	93	31·8	4	1·4	**0·001**
	36–60	282	124	44	28	9·9	123	43·6	7	2·5	
	> 60	65	28	43·1	8	12·3	24	36·9	5	7·7	
Sex	Male	114	23	20·1	8	7·0	67	65·8	16	14·0	**0·740**
	Female	498	94	18·8	24	4·8	314	63·1	66	13·3	
Community	Rural	304	61	20·0	10	3·3	197	64·8	36	11·8	**0·137**
	Urban	320	56	17·5	22	6·9	196	61·3	46	14·4	
	*P*-value										
Lived Poverty Index	Non-deprived	412	79	19·1	25	6·1	258	62·6	50	12·1	**0·396**
	Deprived	195	37	19·0	7	3·6	120	61·5	31	15·9	
Income	< 500	166	29	17·5	6	3·6	110	66·3	21	12·7	**0·441**
	500–1285	200	44	22·0	16	8·0	114	57·0	26	13·0	
	> 1285	70	11	15·7	4	5·7	45	64·3	10	14·2	
Education	None	121	14	11·6	0	0	97	80·2	10	8·3	**< 0·001**
	Primary	63	9	14·3	4	6·3	39	61·9	11	17·5	
	Junior Secondary	190	42	22·1	9	4·7	120	63·0	19	10·0	
	Senior Secondary	167	36	21·6	10	6·0	91	54·5	30	18·0	
	Tertiary	71	16	22·5	9	12·7	34	47·9	12	16·9	

Definition of household food consumption patterns.

High-risk protective: Households consume both high-risk and protective foods frequently.

Low-risk protective: Households consume high-risk foods infrequently and protective foods frequently.

High-risk vulnerable: Households consume high-risk foods frequently and protective foods infrequently.

Low-risk vulnerable: Households consume both high-risk and protective foods infrequently.

Boldface indicates statistically significant results.

In terms of socio-demographic associations, age and education status characterised different household dietary patterns, while income, community type, sex and lived poverty were not. Close to 60 % of households with food purchasers below 36 years of age followed a ‘high obesogenic and high protective’ dietary pattern compared with 44 % of those between 36–60 years and 43 % of those above 60 years category. This indicates that households with younger food decision makers consume both obesogenic and protective foods alike. Only 1·4 % of food decision makers below 36 years, compared with 2·5 % of those between 36–60 years and 7·7 % of those above 60 years followed the ‘high protective and low obesogenic dietary pattern’. This indicates that household frequency of consumption of the healthiest food pattern increased with the age of the food purchaser or food acquisition decision maker.

Likewise, for education, consumption of the ‘high obesogenic and high protective dietary pattern’ increased with education, where just 11 % of households whose food purchasers had no education consumed the category frequently, compared with 14 % for the households with food purchasers with primary education and 22·1 % households of food purchaser with tertiary education (*P* < 0·001). Similarly, consumption of ‘high protective and low obesogenic dietary pattern’ was lowest in households whose food purchaser had no education (8·3 %) and highest in the households whose food purchaser had secondary education (18 %). For all the food purchaser education categories, the most followed dietary pattern was the ‘low obesogenic and low protective dietary pattern’, decreasing with education, from 80·2 % for those with no education and 47·9 % among households whose food purchaser had tertiary education.

### Findings of value chain analysis of obesogenic *v*. protective foods

#### Country of origin and producers of obesogenic *v*. protective foods

**Figure 3.**
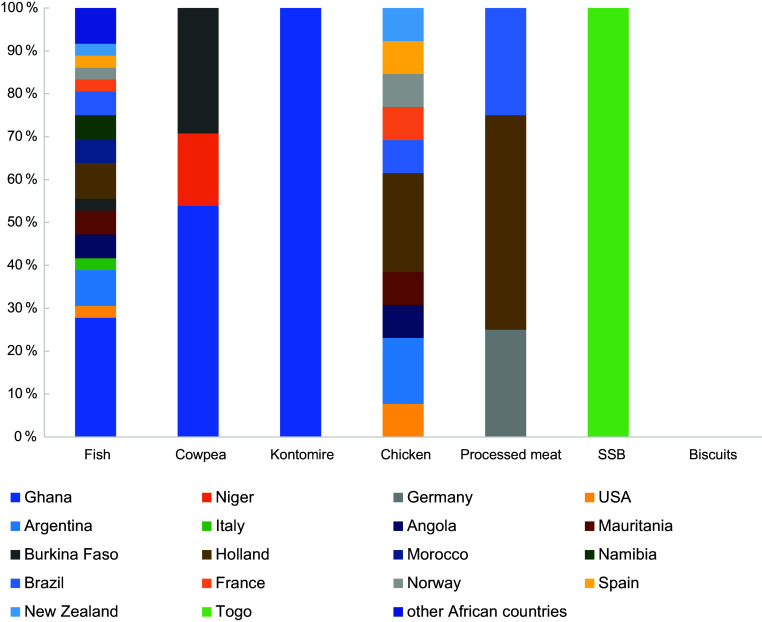
countries from which foods sold by traders are sourced.

Figure 3 illustrates the countries from which retailers and wholesalers sourced their commodities directly. For SSB, only one retailer/wholesaler sourced directly from Togo. Fish was sourced from fifteen other countries apart from Ghana, which spans across the globe. The countries fish was sourced from included Morocco, Niger, Holland, Norway, Spain, New Zealand, USA, Angola, Mauritania, Argentina, Brazil and Namibia. Cowpea was predominantly sourced from within Ghana, as well as from Niger and Burkina Faso, both of which are within Africa. On the other hand, cocoyam leaves (*kontomire*) were exclusively sourced from within Ghana. Biscuit retailers and wholesalers interviewed could not ascertain where their products were produced, although many indicated that they were imported. The protective foods, except fish, generally had a more substantial producer presence in Ghana, while the primary producers of obesogenic foods, such as SSB and processed meat, were largely from countries outside Ghana.

#### Retail of obesogenic *v*. protective food in Ghana

Table 6 shows the characteristics of buyers of selected obesogenic and protective food commodities from retail shops. For the obesogenic food commodities, most buyers who purchased from retailers were female, younger (< 36), had basic education, and were of low- or middle-income status. SSB was the only food commodity that males recorded a higher purchase frequency, while people with higher education often bought processed meat. Middle- and high-income people also bought processed meat more often than low-income individuals. More institutional buyers bought processed meat (20·3 %) than SSB (9 %) and biscuits (0 %), while more households bought biscuits (100 %) than processed meat (57·5 %) and SSB (75·3 %).

**Table 6.** Socio-demographic characteristics of household food consumption decision maker by obesogenic and healthy foods purchase from retail outlets Table 6 long description.

			Income levels (%)			Age (%)			Education level (%)			Sex (%)		Category of buyers (%)		
	Focal Commodity	*n*	Low	Middle	High	< 36 years	36–60 years	> 60 years	Basic	Secondary	Tertiary	Males	Females	Institutional buyers	Other traders	Households
Obesogenic food	Biscuits	209	51·0	39·0	9·0	68·7	28·7	2·7	60·0	32·3	7·7	45·3	54·7	0	0	100·0
	Processed meat	209	29·0	38·0	34·0	38·0	48·5	13·5	28·2	34·8	37·0	30·2	69·8	20·3	22·2	57·5
	SSB	209	43·0	36·0	21·0	55·5	35·3	9·2	38·8	35·9	25·4	52·1	47·9	9·0	15·7	75·3
Protective food	Cowpea	209	34·0	50·0	16·0	39·3	51·7	9·0	41·7	34·4	23·9	25·0	75·0	0	29·7	70·3
	Fish	209	39·0	22·0	39·0	45·7	45·0	9·3	34·8	32·2	33·0	28·6	71·4	2·5	18·2	79·3
	Cocoyam leams (*Kontomire*)	209	59·0	25·0	17·0	42·2	45·7	12·2	51·7	23·3	25·0	20·0	80·0	5·2	16·0	78·8

For protective food commodities, most buyers were female and older (36–60 years age group). Middle-income people often bought cowpeas, while low-income individuals bought cocoyam leaves (*kontomire)* and fish more frequently. High-income people also bought fish more frequently and recorded the same percentage as low-income persons.

#### Distribution/wholesale of obesogenic *v*. protective foods

Table 7 shows the characteristics of buyers of obesogenic *v*. protective food commodities from wholesalers/distributors or those who bought in bulk. Low- to middle-income buyers bought more biscuits (low 39 %, middle 43 %) in wholesale shops than high-income (18·0 %), while high-income buyers bought more processed meat (40 %) than low (27 %) and middle (33 %) income buyers. More institutional buyers bought SSB and processed meat in large volumes than biscuits. Older people bought processed meat, while younger people bought biscuits from distributors. Higher-educated people bought processed meat and SSB more than biscuits, while lower-educated people bought biscuits more than SSB and processed meat from distributors. Institutional buyers purchased more SSB than processed meat from distributors.

**Table 7.** Socio-demographic characteristics of household food consumption decision maker by obesogenic and healthy foods purchase from wholesale/distributor outlets Table 7 long description.

			Income levels (%)			Age group (%)			Education level (%)			Sex (%)		Category of buyers (%)		
	Food commodity	*n*	Low	Middle	High	< 36 years	36–60 years	> 60 years	Basic	Secondary	Tertiary	Males	Females	Institutional buyers	Other traders	Households
Obesogenic foods	Biscuit/cookies	185	39·0	43·0	18·0	49·8	42·0	8·2	39·2	47·1	13·7	26·5	73·5	7·5	85·3	7·2
	Processed meat	185	27·0	33·0	40·0	30·8	50·0	19·2	37·7	36·2	26·2	26·9	73·1	23·1	53·1	23·8
	SSB	185	23·0	35·0	43·0	37·4	48·6	13·9	26·2	38·8	35·0	43·0	56·9	31·9	54·0	14·1
Protective food	Cowpea	185	39·0	47·0	15·0	26·1	63·4	10·5	53·5	40·5	5·9	20·3	79·7	16·3	80·7	3·0
	Fish	185	30·0	40·0	29·0	29·3	58·5	12·2	48·9	35·1	16·0	19·9	80·1	20·0	60·3	19·7
	Cocoyam leaves (*Kontomire*)	185	55·0	32·0	13·0	37·8	49·2	13·0	61·7	32·8	5·5	7·9	92·1	19·3	63·8	17·0

SSB, sugar-sweetened beverage.

Higher-income buyers bought fish in large volumes rather than cowpea and cocoyam leaves for protective food commodities, while lower-income buyers purchased cowpea and cocoyam leaves (*kontomire*). Higher-educated buyers purchased fish, while lower-educated people bought cowpeas. More males bought cowpeas, while more females bought fish and cocoyam leaves *(kontomire)* from distributors.

## Discussion

The ROFE project was undertaken to understand better the drivers of the changing nature of food marketing in urban and rural communities in South Africa and Ghana and the potential policy levers available to improve the healthfulness of the local food environment^(20)^. The project was implemented in three phases and explored household decision-making on food consumption and related factors, retail, distribution/wholesale and production/country of origin of protective *v*. obesogenic food commodities. We utilised a political economy analysis approach to elicit information to inform the development of stronger policies to promote nutrition and support other food system objectives^(20)^. Initial findings indicated that the type of foods available in retail outlets in a neighbourhood drives food consumption and neighbourhoods with more obesogenic food commodities in retail shops consume more obesogenic foods^(21)^.

In the current study, we characterised socio-demographic factors associated with household food consumption, retail and distribution. We found that a large proportion (4 in 10 women) of household food acquisition decision makers were younger women below 36 years and had low education (basic education of nine years) and monthly income. The finding suggests that within the Ghanaian context, decision-making for food intake is generally made by younger people with little formal education and income. Studies have reported that food intake among households with younger decision-makers is associated with a higher intake of obesogenic and less healthy foods^(28,29)^, which is in congruence with the findings of this study. This finding also points out the need for simplified photographic nutrition education campaigns to increase nutrition knowledge and healthy food selection within households.

Using weekly food consumption as frequent and below weekly consumption as infrequent, both SSB and confectionery were consumed more frequently than processed meat across households. Issues around availability and affordability may explain why processed meat consumption is less frequently consumed than SSB. SSB are relatively cheaper obesogenic foods^(30)^ and more common compared with processed meat. The effect of cost and affordability on the consumption of SSB and processed meat supports the fact that policies that increase the price of ‘unhealthy’ foods like SSB^(31)^ can be implemented to reduce their consumption. This justifies the recently enacted SSB tax in Ghana, which is expected to curb SSB consumption through increased production costs and selling prices^(32)^. It is also worth noting that other factors beyond cost and availability, including personal preferences, can affect the consumption frequency of obesogenic foods^(33)^.

The demographic characterisation of households’ consumption of obesogenic foods indicates that age was associated with the consumption of all the analysed foods. Age was associated with SSB, processed meat and confectionery consumption. Sex was only associated with SSB consumption. Education and urbanisation were associated with SSB and confectionery consumption. Several studies have demonstrated the linkages between socio-demographic factors and obesogenic food consumption^(34–36)^. Epidemiological studies have documented that ultra-processed foods appeal mostly to young people, who are more likely to disregard traditional foods^(37,38)^. It is unclear whether retailers and distributors of obesogenic foods target persons with these demographic characteristics or if they prefer obesogenic foods. Either way, the findings suggest that just as social behaviour change communication can target persons within this demography are more likely to consume these foods, interventions should also focus on making the food environment less obesogenic^(38)^.

Issues around access can also explain the consumption differences observed between urban and rural households in this study. SSB have been reported to be more accessible in urban areas^(39)^, and this may explain why urban households in this study consumed more SSB than people living in rural areas. For example, legumes are more expensive and require more fuel to cook^(40)^ than green leafy vegetables like cocoyam leaves (*Kontomire),* and as such, non-deprived households may consume them more frequently than deprived households. Fish is commonly consumed across the country, and though the consumption frequency may be higher, quantities consumed may need to be better. In this study, we focused on frequency and not quantity.

Our analysis of patterns of household consumption was used to group households into four obesogenic consumption patterns: those that consumed both obesogenic and protective foods above the cut-offs (high obesogenic and protective dietary pattern), those that consumed obesogenic and protective foods below the cut-offs (low obesogenic and protective pattern), those that consumed obesogenic above the cut-offs and protective foods below the cut-offs (high obesogenic low protective dietary pattern) and those that consumed protective foods above the cut-offs and obesogenic below the cut-offs (high protective low obesogenic dietary pattern). Using this classification, the ‘high protective low obesogenic dietary pattern’ can be considered ‘most healthy’ of the four patterns because households consumed enough protective foods and little obesogenic foods. The worst pattern could be viewed as the ‘high obesogenic low protective dietary pattern’ as households consumed obesogenic foods above the cut-off and protective foods below the cut-off. The ‘high obesogenic and protective dietary pattern’ and ‘low obesogenic and protective dietary pattern’ households are also considered vulnerable. For the former group, the benefits of the protective foods could be nullified by the obesogenic foods. At the same time, the latter households may have no protection even though they did not have a high frequency of consumption of obesogenic foods to pose risks.

Our analysis revealed that very low proportions of households followed the ‘most protective’ and ‘least protective’ pattern. Together, <20 % of households followed these two patterns. This implies that most of the households were vulnerable by consuming both obesogenic and protective foods frequently or infrequently, respectively. In Zambia, a study reported that two-thirds of households used modern and traditional retailers simultaneously, and both groups of retailers drove the consumption of ultra-processed and healthy foods^(41)^. It shows that simultaneous consumption of obesogenic and healthy foods is common in Sub-Sahara Africa and driven by both modern and traditional outlets. Our findings also imply that households did not discriminate between obesogenic and protective foods, consuming them alike. It is possible that access is the driver of consumption, so policies that regulate access to obesogenic foods to reduce consumption should be considered. The global cost of NCD is projected to reach $47 trillion, and the high number of deaths from NCD^(5)^ makes our finding that most Ghanaians consume obesogenic foods and protective foods indiscriminately worrying.

Our analysis of the association between household food intake patterns and socio-demographic status revealed some associations. Overall, consumption of the most protective pattern increased with age and education. Households with older and more educated persons in charge of food acquisition consumed this pattern than households with less educated and younger persons in charge of food acquisition. Consumption of SSB has been shown to decrease with age and education in other studies^(42)^. More educated household food purchasers may know the benefits of healthy eating or probably have better incomes to afford such dietary patterns.

The findings of the value chain analysis agreed with the household consumption study in many regards. From the retailers’ perspective, buyers of the obesogenic foods, except processed meat, were younger and of the lower third of income groups, as observed for the consumption study. For the protective foods, on the other hand, buyers were older and leaned towards the upper-income groups, except for cocoyam leaves (*Kontomire)*. Cocoyam leaves (*Kontomire*) are a green leafy vegetable that is cheaper and more affordable than fish and cowpea. Also, processed meat is more expensive than SSB. From the perspectives of wholesalers, income seemed to drive the type of purchase, and high-income buyers bought more costly foods in bulk, whether protective or obesogenic. It suggests that cost is a big driver of obesogenic and protective foods consumption, retail and distribution. It has been reported that even though the preference of Ghanaians for imported fatty meat is low, and they consider local versions less fatty, healthier and more preferred^(33)^, consumption is driven by cost and affordability^(33)^. Our findings seem to resonate with this earlier study.

As far as the origin of obesogenic and protective foods in the Ghanaian market is concerned, the majority of the obesogenic foods were sourced from outside Ghana, while the protective foods have their production mainly within Ghana. For instance, two obesogenic commodities, SSB and processed meat, were sourced entirely from outside the country, while the legumes and vegetables were entirely sourced from Ghana and fish from both within and outside Ghana. This creates a course for concern. Currently, developing countries account for most of the increases in the sales of ultra-processed foods, which are continuously being marketed aggressively on the Africa continent^(37)^. This occurrence is also fuelled by trade liberalisation^(41,42)^ making trade liberalisation a driver of obesogenic food spread, access and consumption in many LMIC. Regulatory measures to curb the influx of obesogenic foods into the country should be implemented. To curb the increasing rate of CVD, Ghana implemented a fatty meat policy in the 1990s that specifies fat cut-offs for meat and other animal products^(33)^. The policy was reported to have helped reduce the availability of low-quality high-fat meats, especially turkey tails and chicken feet, and this shows the ability to use standards to reduce availability and access to unhealthy food. Strengthening the implementation and enforcement of public health policies in the country should be encouraged from lessons of the fatty meat policy.

The ROFE study has some limitations. The household food consumption study was undertaken using FFQ. The data on consumption are, therefore, on frequency of consumption and not indicative of amounts or volumes of consumption. The data on consumption were provided by the household food purchaser and consumption decision maker and socio-demographic data like education, age and sex were of this person. This limits the socio-demographic inferences to be made as it is possible that other demography within the household may behave differently. In this study, only the three most consumed obesogenic and healthy foods were analysed further, which could have limited the analysis. However, prioritising the most consumed foods was to help uncover the most significant factors to household food consumption and to thoroughly explore the key issues rather than everything. This has facilitated a more nuanced comprehension of associated factors for consuming obesogenic *v*. protective foods by households. Also, the study was undertaken in just one rural and urban community in Ashanti Region, Ghana, and this limits the generalisability of our findings.

To determine the patterns of household intake of obesogenic *v*. protective foods, the research team agreed on three foods for the protective group at a consumption frequency of five or more times per week as the cut-off for protective, while the obesogenic foods cut-off was four foods at two or more weekly consumption frequency. This allowed a dichotomous categorisation for each group and thus four patterns. The choice on number and frequency was based on the variations in number of protective and obesogenic foods on the FFQ. There may be some limitations, and this approach needs further variation, but the determination of food consumption patterns was not completely arbitrary. It was based on some literature and careful deliberations by the research team. The research team also has significant experience of the context though the approach may require further validation.

In conclusion, the soaring overweight/obesity and associated NCD currently observed in Ghana seem driven by an obesogenic food environment, consumption and value chain. Our study revealed that households in both urban and rural Ghana consumed both obesogenic and healthy foods alike and very few followed a strictly healthy food intake pattern. The value chain analysis suggests that younger, urban and less educated food purchaser households consumed more obesogenic foods. This is not surprising as these foods will be more available among urban dwellers, appeal better to younger people, while less educated urban dwellers are likely less informed about good nutrition and/or may have lower access to healthier options. This may, however, not apply to the entire Ghanaian population as a results of limitations inherent in the methods employed for the study. Ultimately, almost all the obesogenic foods have their source outside the country, and this gives clear benefits for formulating and enforcing policies that regulate the importation of unhealthy foods to promote and protect public health.

## Table 1 Long description

A table categorizing foods into obesogenic and protective based on processing levels. The table has two columns: Categories and Definition and description. The Categories column lists two types: Obesogenic foods and Protective foods. The Definition and description column provides details for each category. For Obesogenic foods, it lists ultra-processed, processed, and fried foods and ingredients, including examples like processed meat, instant noodles, sweets, and commercial bread. For Protective foods, it lists minimally processed, plant-based foods, including examples like fresh and cooked vegetables, fruits, legumes, and fish.

Navigate back to Table 1.

## Table 2 Long description

A table categorizing household food consumption patterns based on frequency of protective and obesogenic food intake. The table has two columns: Categories and Definition and description. The Categories column lists four types of dietary patterns: Consumption of protective foods by households, Consumption of obesogenic foods by households, High obesogenic and high protective pattern, High protective and low obesogenic pattern, High obesogenic and low protective pattern, Low obesogenic and low protective dietary pattern. The Definition and description column provides definitions for each category. For example, Consumption of protective foods by households is defined as frequently consuming 5 or more times per week for three of the protective foods. Consumption of obesogenic foods by households is defined as frequently consuming 2 or more times per week for four of the obesogenic foods. The table also defines four dietary patterns based on the frequency of consumption of protective and obesogenic foods.

Navigate back to Table 2.

## Table 3 Long description

The table presents socio-demographic characteristics of household food consumption decision makers. It has six columns: Variable, Frequency, and Percentage. The variables include Age in years, Sex, Community, Lived poverty index, Income, and Education. Row 1: Age in years, Frequency 248, Percentage 40.6. Row 2: Age in years 36-60, Frequency 267, Percentage 43.7. Row 3: Age in years greater than 60, Frequency 96, Percentage 15.7. Row 4: Sex Male, Frequency 114, Percentage 18.6. Row 5: Sex Female, Frequency 498, Percentage 81.4. Row 6: Community Rural, Frequency 304, Percentage 48.7. Row 7: Community Urban, Frequency 320, Percentage 51.3. Row 8: Lived poverty index Non-deprived, Frequency 412, Percentage 67.9. Row 9: Lived poverty index Deprived, Frequency 195, Percentage 32.1. Row 10: Income less than 500, Frequency 166, Percentage 38.1. Row 11: Income 500-1265, Frequency 200, Percentage 45.9. Row 12: Income greater than 1265, Frequency 70, Percentage 16.1. Row 13: Education None, Frequency 121, Percentage 19.8. Row 14: Education Primary, Frequency 63, Percentage 10.3. Row 15: Education Junior Secondary, Frequency 190, Percentage 31.0. Row 16: Education Senior Secondary, Frequency 167, Percentage 27.3. Row 17: Education Tertiary, Frequency 71, Percentage 11.6.

Navigate back to Table 3.

## Table 4 Long description

The table presents the proportion of households consuming specific food groups weekly, categorized by various socio-demographic factors. It includes columns for SSB, processed meat, confectionery, cooked vegetables, legumes, and fish, with overall proportions and breakdowns by age, sex, community, LPI, income, and education. Each row provides specific percentages for these categories. Notable trends include higher consumption of SSB and confectionery among younger age groups and those with higher income, while cooked vegetables and fish are more commonly consumed by older age groups and those in urban areas.

Navigate back to Table 4.

## Table 5 Long description

The table compares household dietary intake patterns by four different patterns: high protective and high obesogenic, high obesogenic and low protective, low obesogenic and low protective, and high protective and low obesogenic. The table includes variables such as age, sex, community, lived poverty index, income, and education. It provides the total number and percentage of households in each category for these variables. Notable trends include a higher percentage of households with high protective and high obesogenic dietary patterns among those under 36 years old, and a higher percentage of households with low obesogenic and low protective dietary patterns among those over 60 years old. The table also shows that the majority of households are not on the extreme ends of following mainly protective or obesogenic dietary patterns.

Navigate back to Table 5.

## Table 6 Long description

The table compares the characteristics of buyers of selected obesogenic and protective food commodities from retail shops. It has 8 columns and 6 rows, including headers. The columns are Focal Commodity, n, Income levels (%), Age (%), Education level (%), Sex (%), and Category of buyers (%). The rows are labeled with different food commodities: Biscuits, Processed meat, SSB, Cowpea, Fish, and Cocoyam leaves (Kontomire). Each row provides data on the number of buyers (n), income levels (Low, Middle, High), age groups (< 36, 36-60, > 60 years), education levels (Basic, Secondary, Tertiary), sex (Males, Females), and category of buyers (Institutional buyers, Other traders, Households). For example, Row 1: Biscuits, 209, Low 51.0, Middle 39.0, High 9.0, < 36 68.7, 36-60 28.7, > 60 2.7, Basic 60.0, Secondary 32.3, Tertiary 7.7, Males 45.3, Females 54.7, Institutional buyers 0, Other traders 0, Households 100.0.

Navigate back to Table 6.

## Table 7 Long description

The table has 185 rows and 12 columns. The columns are Food commodity, n, Income levels (Low, Middle, High), Age group (< 36 years, 36-60 years, > 60 years), Education level (Basic, Secondary, Tertiary), Sex (Males, Females), and Category of buyers (Institutional buyers, Other traders, Households). The rows are labeled with different food commodities: Biscuit/cookies, Processed meat, SSB, Cowpea, Fish, and Cocoyam leaves (Kontomire). Row 1: Biscuit/cookies, 185, Income levels (Low 39.0, Middle 43.0, High 18.0), Age group (< 36 years 49.8, 36-60 years 42.0, > 60 years 8.2), Education level (Basic 39.2, Secondary 47.1, Tertiary 13.7), Sex (Males 26.5, Females 73.5), Category of buyers (Institutional buyers 7.5, Other traders 85.3, Households 7.2). Row 2: Processed meat, 185, Income levels (Low 27.0, Middle 33.0, High 40.0), Age group (< 36 years 30.8, 36-60 years 50.0, > 60 years 19.2), Education level (Basic 37.7, Secondary 36.2, Tertiary 26.2), Sex (Males 26.9, Females 73.1), Category of buyers (Institutional buyers 23.1, Other traders 53.1, Households 23.8). Row 3: SSB, 185, Income levels (Low 23.0, Middle 35.0, High 43.0), Age group (< 36 years 37.4, 36-60 years 48.6, > 60 years 13.9), Education level (Basic 26.2, Secondary 38.8, Tertiary 35.0), Sex (Males 43.0, Females 56.9), Category of buyers (Institutional buyers 31.9, Other traders 54.0, Households 14.1). Row 4: Cowpea, 185, Income levels (Low 39.0, Middle 47.0, High 15.0), Age group (< 36 years 26.1, 36-60 years 63.4, > 60 years 10.5), Education level (Basic 53.5, Secondary 40.5, Tertiary 5.9), Sex (Males 20.3, Females 79.7), Category of buyers (Institutional buyers 16.3, Other traders 80.7, Households 3.0). Row 5: Fish, 185, Income levels (Low 30.0, Middle 40.0, High 30.0), Age group (< 36 years 29.3, 36-60 years 58.5, > 60 years 12.2), Education level (Basic 48.9, Secondary 35.1, Tertiary 16.0), Sex (Males 19.9, Females 80.1), Category of buyers (Institutional buyers 20.0, Other traders 60.3, Households 19.7). Row 6: Cocoyam leaves (Kontomire), 185, Income levels (Low 55.0, Middle 32.0, High 13.0), Age group (< 36 years 37.8, 36-60 years 49.2, > 60 years 13.0), Education level (Basic 61.7, Secondary 32.8, Tertiary 5.5), Sex (Males 7.9, Females 92.1), Category of buyers (Institutional buyers 19.3, Other traders 63.8, Households 17.0).

Navigate back to Table 7.
